# Association between Depressive Symptoms and Bone Stiffness Index in Young Adults: The Kangwha Study

**DOI:** 10.1371/journal.pone.0069929

**Published:** 2013-07-24

**Authors:** Sun Min Oh, Hyeon Chang Kim, Kyoung Min Kim, Song Vogue Ahn, Dong Phil Choi, Il Suh

**Affiliations:** 1 Department of Preventive Medicine, Yonsei University College of Medicine, Seoul, Republic of Korea; 2 Department of Internal Medicine, Institute of Endocrine Research, Yonsei University College of Medicine, Seoul, Republic of Korea; 3 Department of Preventive Medicine, Wonju College of Medicine, Yonsei University, Wonju, Republic of Korea; China Medical University, Taiwan

## Abstract

**Objective:**

Young adulthood is an important period for both bone and mental health. This study investigated the association between depressive symptoms and bone density in apparently healthy Korean men and women aged 29−32 years.

**Methods:**

This study is a cross-sectional analysis of data from 123 men and 133 women who completed follow-up examinations of the Kangwha study in 2010−2011. Bone stiffness index (SI) was measured at the os calcis using a quantitative ultrasound device. Depressive symptoms were evaluated using the Korean version of the Beck Depression Inventory (K-BDI) and classified as normal (K-BDI <10), mild (K-BDI 10–15), and moderate to severe (K-BDI ≥16).

**Results:**

Moderate to severe depressive symptoms were prevalent among 11.4% of men and 19.6% of women. Higher K-BDI scores were significantly correlated to SI in men, before (ρ = –0.286, *p* = 0.001) and after (ρ = –0.228, *p* = 0.013) adjustment for covariates. Men with depressive symptoms tended to have a lower SI; multivariate-adjusted mean SI in men with normal, mild, and moderate to severe depressive symptoms was 104.1±3.1, 100.9±5.9, and 94.1±7.8, respectively (*p* for trend = 0.021). In contrast, no significant correlations were identified in women.

**Conclusions:**

Depressive symptoms were significantly associated with lower SI in men, but not in women. Further studies are necessary to evaluate the impact of depression on developing osteoporosis or osteoporotic fractures later in life.

## Introduction

Osteoporosis is characterized by low bone mass and microarchitectural deterioration of bone tissue, with a consequent increase in bone fragility and susceptibility to fracture [1]. Osteoporosis is usually asymptomatic, but osteoporotic fracture can cause considerable health care burden due to hospitalization, limited mobility, and significant mortality. Osteoporosis is a prevalent disease, afflicting over 200 million patients worldwide, and prevalence increases as the population ages [2]. Consequently, health care costs are estimated to be doubled by 2050 [2]. Bone mass increases to peak until 20 to 30 years of age and generally decreases with age [3]. Achieving high peak bone mass in young adulthood is important because it predicts a relatively higher bone mass and a lower osteoporotic fracture incidence in later life [3]. Young adulthood is also an important period for mental health; between 20 and 30 years of age is the most common time of depression onset [4]. In the World Mental Health Survey, the mean age for the onset of depression was 28.9 years in 10 developed countries and 27.2 years old in developing countries [4]. Depression is also common mental disorder; at least 350 million people suffered from depression worldwide [5]. Furthermore, depression is the leading cause of disability because it can become chronic or recurrent, substantially impairing an individuals’ quality of life [5].

Interestingly, since a case-control study suggested an inverse association between major depressive disorder (MDD) and lumbar bone mineral density (BMD) [6], the link between depression and BMD has been continuously studied. Recently, systematic reviews and meta-analyses of epidemiologic studies demonstrated that MDD was associated with lower BMD [7]–[9]. Specifically, in a systematic review, 76% (25 out of 33) of the qualified articles reported an inverse association between depression or depressive symptoms and BMD at the AP spine, femoral neck, and total femur [9]. In relation to areal BMD at the forearm, a large scale community study with 1,194 men and 7,842 women demonstrated a negative association between depressive symptoms and BMD in men and heavier women in a cross-sectional analysis [10]. However, previous studies were performed mainly in middle-aged or older white populations. For young adults, limited information is available and the results are inconsistent. In a nationwide study with various ethnic groups in the U.S., MDD or dysthymia was associated with lower BMD in men, but not in women [11]. In addition, several studies in premenopausal women showed either inverse associations between depression and BMD or no significant associations [7]–[9]. The inconsistent results may be due to small sample size, differences in study design, use of different assessment tools for depression and BMD, or evaluating different ethnic groups. Still, little evidence is available in young Asian men and women. Therefore, the current study aimed to investigate the association between depressive symptoms and bone density in apparently healthy Korean men and women aged 29 to 32 years.

## Materials and Methods

### Study Participants

The Kangwha Study is a community-based prospective cohort study which began in 1986 with 6-year-old school children in Kangwha County located on the West coast of South Korea. Details of this study were previously described [12], [13]. BMD measurements have been added in 2010; therefore, the present study is a cross-sectional analysis of data from follow-up examinations in 2010 and 2011. Among 123 men and 141 women aged 29 to 32 years, eight participants were excluded from the present analyses due to at least one of the following reasons: absence of bone density measurement (n = 1), previously diagnosed depression (n = 3), missing blood tests (n = 1), or unknown age at menarche (n = 6). The participants diagnosed with depression were excluded to avoid effects of antidepressants or other lifestyle changes due to the known depression. Additionally, no participant reported to have been diagnosed cancer, stroke, ischemic heart disease, or osteoporosis. Finally, 256 participants (123 men and 133 women) were eligible for this study. All participants in this survey provided written informed consent. This study was approved by the Institutional Review Board of Yonsei University Health System (4–2009–0624) and monitored by the Human Research Protection Center of Severance Hospital, Yonsei University Health System.

### Measurements

Trained research staffs explained the study questionnaires to participants and asked questions pertaining medical history, health behavior, and other health-related information. Anthropometrics and blood pressure were measured according to the pre-developed protocol. Body mass index (BMI) was calculated as weight in kilograms divided by height in meters squared. Smoking status was categorized as either current smoker versus non-smoker (past or never). At-risk drinking was defined as consuming an average of more than 2 drinks/day for men and 1 drink/day for women. Blood samples were collected after an 8-hour fasting period and handled according to standard procedures. Bone stiffness index (SI) at the os calcis was measured using a quantitative ultrasound (QUS) device, the Achilles Express ultrasonometer (GE Medical Systems LUNAR, WI, USA). SI was calculated using the values of the velocity and the frequency-dependent attenuation of the sound waves; SI = (0.67×broadband ultrasound attenuation in dB/MHz)+(0.28×speed of sound in m/sec) –420. Although the QUS method is an alternative tool to evaluate bone density, the GE Lunar Achilles that we used in this study has been proven in clinical studies and the SI value is considered to be clinically useful [14]. Quality assurance was confirmed using a phantom every week according to the protocol. Precision for the QUS phantom was comparable to that of dual energy X-ray absorptiometry (DXA) with a coefficient variation of SI of around 2% [15].The Korean version of the Beck Depression Inventory (K-BDI) was used to assess depressive symptoms. The BDI is a widely used screening tool for depression that consists of 21 items assessing symptoms and attitudes more frequently observed in depressed patients [16], [17]. Each item can be rated from 0 to 3 in terms of intensity, and higher scores reflect more severely depressed mood. The K-BDI is a translated and validated version of the BDI for use in Korean population [18], [19]. Depressive symptoms were classified as normal (K-BDI <10), mild (K-BDI 10−15), and moderate to severe (K-BDI ≥16) [19].

### Statistical Analysis

Among the baseline characteristics measured, age, anthropometrics, metabolic factors, lifestyle factors, and female reproductive factors that might be related to either bone density or depressive symptoms were selected [1], [5]. All analyses were performed for men and women separately because most baseline characteristics differed by sex. Baseline characteristics according to depressive symptoms (classified as normal, mild, and moderate to severe) were compared using analysis of variance for continuous variables with normal distribution, Kruskal-Wallis test for continuous variables with skewed distribution, and chi-square test for categorical variables. To investigate whether the mean SI was significantly different between classified depressive symptoms, Tukey’s test was performed. Correlations of the SI and covariates were analyzed using Spearman’s (partial) correlation analyses. Potential confounders were selected using a stepwise regression analysis and clinically important variables were retained even if they did not reach a significant level of association. Selected covariates were age, BMI, at risk drinking (>2 drinks/day), and regular exercise (≥1/week) in men and age, BMI, at risk drinking (>1 drink/day), age at menarche, and duration of breastfeeding (0, 1−11, ≥12 months) in women. A series of unadjusted and multivariate-adjusted analysis of covariance were performed to calculate adjusted means and 95% confidence intervals of the SI according to depressive symptoms. Multivariate-adjusted regression analysis was performed to yield adjusted *R*-square to investigate ability of the covariates to explain the SI. No significant *multicollinearity was confirmed among investigated variables by computing a* variance inflation factor. A *p*-value less than 5% was considered significant. All statistical analyses were performed using the SAS software package (version 9.2.1; SAS Institute, Cary, NC, USA).

## Results

In the present study population, mean ages of men and women were 30.8±0.6 (ranging 29.1 to 32.2) and 30.7±0.6 (ranging 29.8 to 32.1) years old, respectively. The percentage of participants with normal, mild, and moderate to severe depressive symptoms was 69.1% (n = 85), 19.5% (n = 24), and 11.4% (n = 14), respectively, in men (Table 1) and 54.9% (n = 73), 25.6% (n = 34), and 19.6% (n = 26), respectively, in women (Table 2). The SI of participants with moderate to severe depressive symptoms was significantly lower than that of normal participants in men, but not in women. Other characteristics of men and women are summarized in Table 1 and Table 2, respectively.

**Table 1:** Baseline characteristics in men aged 29−32 years according to depressive symptoms^a^.

	Normal	Mild	Moderate to severe	*p*-value
	(n = 85, 69.1%)	(n = 24, 19.5%)	(n = 14, 11.4%)	
K-BDI score	4 [2], [7]	12.5 [10.0, 13.5]	18 [17], [21]	<0.001^b^
Age (years)	30.8±0.6	30.8±0.6	30.9±0.6	0.741^c^
Weight (Kg)	73.1±10.8	75.0±8.9	71.7±8.9	0.598^c^
Height (cm)	173.6±5.3	174.3±5.3	173.4±4.3	0.826^c^
Body mass index (Kg/m^2^)	24.3±3.4	24.7±3.2	23.8±2.3	0.670^c^
Systolic blood pressure (mmHg)	124.0±12.1	129.2±12.3	123.4±10.4	0.152^c^
Diastolic blood pressure (mmHg)	73.5±7.8	76.9±7.3	76.0±10.1	0.140^c^
Laboratory tests				
Total cholesterol (mg/dL)	188.2±34.2	186.9±36.3	194.6±36.6	0.788^c^
HDL cholesterol (mg/dL)	45.4±9.0	45.7±9.0	46.4±9.4	0.928^c^
Triglycerides (mg/dL)	96 [69, 148]	120 [79, 168]	82.5 [72.0, 140.0]	0.431^b^
Fasting plasma glucose (mg/dL)	94.4±9.3	95.5±7.7	89.6±8.3	0.127^c^
HbA1c (%)	5.5±0.3	5.4±0.2	5.5±0.3	0.373^c^
Insulin (µIU/mL)	7.9 [6.5, 10.7]	7.0 [6.2, 9.6]	7.2 [5.6, 9.0]	0.323^b^
hsCRP (mg/L)	0.4 [0.2,1.0]	0.9 [0.4, 2.0]	0.6 [0.3, 2.1]	0.086^b^
Current smoking	44 (51.8)	8 (33.3)	10 (71.4)	0.069^d^
At-risk drinking^e^	36 (42.4)	12 (50.0)	9 (64.3)	0.289^d^
Regular exercise (≥1/week)	32 (37.7)	6 (25.0)	5 (35.7)	0.517^d^
Bone stiffness index	104.5±14.3	100.2±12.1	93.2±20.8	0.030^c^

Data are expressed as median [interquartile range], mean ± standard deviation, or N (%).

K-BDI, the Korean version of the Beck Depression Inventory; HDL, high-density lipoprotein; hsCRP, high-sensitivity C-reactive protein.

a Depressive symptoms were classified as normal (K-BDI <10), mild (K-BDI 10−15), and moderate to severe (K-BDI ≥16).

b Kruskal-Wallis test for continuous variables with skewed distribution;

c Analysis of variance for continuous variables with normal distribution;

d Chi-square test for categorical variables were performed to compare baseline characteristics according to depressive symptoms (classified as normal, mild, and moderate to severe).

e At-risk drinking was defined as consuming an average of more than 2 drinks/day.

**Table 2:** Baseline characteristics in women aged 29−32 years according to depressive symptoms^a^.

	Normal	Mild	Moderate to severe	*p*-value
	(n = 73, 54.9%)	(n = 34, 25.6%)	(n = 26, 19.6%)	
K-BDI score	5 [3], [7]	12.5 [10.0, 14.0]	24 [18], [29]	<0.001^b^
Age (years)	30.7±0.5	30.8±0.6	30.8±0.6	0.692^c^
Weight (Kg)	56.4±7.9	57.2±11.1	56.3±13.2	0.918^c^
Height (cm)	160.4±4.9	160.8±4.2	160.1±4.4	0.839^c^
Body mass index (Kg/m^2^)	21.9±2.9	22.1±3.9	21.9±4.4	0.967^c^
Systolic blood pressure (mmHg)	108.5±12.3	110.0±10.2	107.6±9.5	0.679^c^
Diastolic blood pressure (mmHg)	65.4±9.1	68.1±7.8	66.9±6.2	0.272^c^
Laboratory tests				
Total cholesterol (mg/dL)	179.1±36.1	184.1±24.1	174.9±27.9	0.540^c^
HDL cholesterol (mg/dL)	54.8±12.7	54.4±13.1	52.6±13.9	0.752^c^
Triglycerides (mg/dL)	71 [52, 88]	63.5 [54.0, 84.0]	66.5 [58.0, 98.0]	0.590^b^
Fasting plasma glucose (mg/dL)	88.1±9.9	90.9±12.3	90.0±7.2	0.363^c^
HbA1c (%)	5.4±0.3	5.5±0.3	5.5±0.3	0.411^c^
Insulin (µIU/mL)	7.6 [6.1, 8.8]	7.6 [6.2, 8.9]	7.9 [6.8, 9.8]	0.367^b^
hsCRP (mg/L)	0.3 [0.2, 0.7]	0.3 [0.1, 0.8]	0.4 [0.2, 0.8]	0.405^b^
Current smoking	5 (6.9)	1 (2.9)	7 (26.9)	0.004^d^
At-risk drinking^e^	10 (13.7)	5 (14.7)	7 (26.9)	0.281^d^
Regular exercise (≥1/week)	20 (27.4)	6 (17.7)	5 (19.2)	0.464^d^
Bone stiffness index	94.8±14.4	94.4±11.5	88.4±14.1	0.115^c^
Age of menarche	13.2±1.5	12.8±1.6	13.3±1.2	0.338^c^
Pregnancy (ever)	42 (57.5)	17 (50.0)	17 (65.4)	0.488^d^
No. of children				
None	37 (50.7)	19 (55.9)	11 (42.3)	0.519^d^
1 − 2	24 (32.9)	8 (23.5)	7 (26.9)	
≥3	12 (16.4)	7 (20.6)	8 (30.8)	
Duration of breastfeeding				
None	38 (52.0)	20 (58.8)	12 (46.1)	0.680^d^
1 − 11 months	21 (28.8)	8 (23.5)	6 (23.1)	
≥12 months	14 (19.2)	6 (17.7)	8 (30.8)	
Oral contraceptives				
Never	54 (74.0)	25 (73.5)	16 (61.5)	0.642^d^
Past users	17 (23.3)	9 (26.5)	9 (34.6)	
Current users	2 (2.7)	0 (0.0)	1 (3.9)	

Data are expressed as median [interquartile range], mean ± standard deviation, or N (%).

K-BDI, the Korean version of the Beck Depression Inventory; HDL, high-density lipoprotein; hsCRP, high-sensitivity C-reactive protein.

a Depressive symptoms were classified as normal (K-BDI <10), mild (K-BDI 10−15), and moderate to severe (K-BDI ≥16).

b Kruskal-Wallis test for continuous variables with skewed distribution;

c Analysis of variance for continuous variables with normal distribution;

d Chi-square test for categorical variables were performed to compare baseline characteristics according to depressive symptoms (classified as normal, mild, and moderate to severe).

e At-risk drinking was defined as consuming an average of more than 1 drink/day.

Table 3 shows the correlations between the SI and covariates. In men, the SI had a significantly negative correlation with K-BDI scores in before (ρ = –0.286, *p* = 0.001) and after (ρ = –0.228, *p* = 0.013) adjustment for potential confounders. In addition, regular exercise was positively correlated to the SI (ρ = 0.190, *p* = 0.035). In women, participants with a higher SI tended to have a lower K-BDI score, but the correlation was statistically insignificant. Rather, the SI was negatively correlated with age (ρ = –0.240, *p* = 0.005), at-risk drinking (ρ = –0.173, *p* = 0.047), age of menarche (ρ = –0.259, *p* = 0.003), and the number of children (ρ = –0.195, *p* = 0.025).

**Table 3:** Correlations of bone stiffness index with covariates in men and women aged 29−32 years.

	Men (n = 123)		Women (n = 133)	
	ρ	*p*-value	ρ	*p*-value
Age (years)	−0.028	0.755	−0.240	0.005
Body mass index (Kg/m^2^)	0.043	0.633	0.120	0.171
Systolic blood pressure (mmHg)	0.004	0.964	0.104	0.232
Diastolic blood pressure (mmHg)	−0.054	0.553	−0.003	0.975
Laboratory tests				
Total cholesterol (mg/dL)	−0.145	0.109	0.125	0.152
HDL cholesterol (mg/dL)	−0.007	0.935	−0.006	0.944
Triglycerides (mg/dL)	−0.105	0.247	−0.045	0.605
Fasting plasma glucose (mg/dL)	0.083	0.361	−0.012	0.889
HbA1c (%)	0.008	0.930	0.060	0.492
Insulin (µIU/mL)	−0.028	0.760	0.042	0.631
hsCRP (mg/L)	0.128	0.158	−0.014	0.877
Current smoking	0.088	0.333	−0.007	0.934
At-risk drinking^a^	−0.157	0.084	−0.173	0.047
Regular exercise (≥1/week)	0.190	0.035	0.126	0.148
Age of menarche			−0.259	0.003
No. of children^b^			−0.195	0.025
Duration of breastfeeding^c^			−0.164	0.060
K-BDI score (unadjusted)	−0.286	0.001	−0.148	0.089
K-BDI score(multivariate-adjusted^d^)	−0.228	0.013	−0.118	0.184

K-BDI, the Korean version of the Beck Depression Inventory; HDL, high-density lipoprotein; hsCRP, high-sensitivity C-reactive protein.

a At-risk drinking was defined as consuming an average of more than 2 drinks/day for men and 1 drink/day for women.

b The number of children was classified into 0, 1−2, and ≥3.

c Duration of breastfeeding was classified into 0, 1−11, and ≥12 months.

d Adjusted for age, body mass index, at risk drinking (>2 drinks/day), and regular exercise (≥1/week) in men and age, body mass index, at risk drinking (>1 drink/day), age at menarche, and duration of breastfeeding (0, 1−11, and ≥12 months) in women.

Men with depressive symptoms tended to have a lower SI. The mean SI in men with normal, mild, and moderate to severe depressive symptoms was 104.5±3.2, 100.2±6.0, and 93.2±7.8, respectively (*p* for trend = 0.009), for unadjusted values, and 104.1±3.1, 100.9±5.9, and 94.1±7.8, respectively (*p* for trend = 0.021), for multivariate-adjusted values. In contrast, although the negative trend was observed in an unadjusted model, the statistical significance was attenuated after adjusting for potential confounders in women (Table 4). Factors associated with the SI were demonstrated in Table 5. In men, moderate to severe depressive symptoms (*β* = −9.993, *p* = 0.021) and regular exercise (*β* = 6.185, *p* = 0.029) were significantly associated with the SI. With other covariates, they explained approximately 7% of the SI. In women, age (*β* = −5.757, *p* = 0.004) and age at menarche (*β* = −2.532, *p* = 0.001) were significantly associated with the SI. Together with other covariates, 16% of the variability of the SI was explained.

**Table 4:** Bone stiffness index in men and women aged 29−32 years according to severity of depressive symptoms.

		Men (n = 123)			Women (n = 133)	
	N	Unadjusted SI	Multivariate-adjusted SI^a^	N	Unadjusted SI	Multivariate-adjusted SI^b^
Depressive symptoms^c^						
Normal	85	104.5±3.2	104.1±3.1	73	94.8±3.2	94.6±2.9
Mild	24	100.2±6.0	100.9±5.9	34	94.4±4.6	93.5±4.3
Moderate to severe	14	93.2±7.8	94.1±7.8	26	88.4±5.3	90.1±5.0
*p* for trend		0.009	0.021		0.043	0.128

SI, bone stiffness index; K-BDI, the Korean version of the Beck Depression Inventory.

a Adjusted for age, body mass index, at risk drinking (>2 drinks/day), and regular exercise (≥1/week).

b Adjusted for age, body mass index, at risk drinking (>1 drink/day), age at menarche, and duration of breastfeeding (0, 1−11, and ≥12 months).

c Depressive symptoms were classified as normal (K-BDI <10), mild (K-BDI 10−15), and moderate to severe (K-BDI ≥16).

**Table 5:** Factors associated with bone stiffness index in men and women aged 29−32 years.

	Men (n = 123)			Women (n = 133)		
	PE	SE	*p*-value	PE	SE	*p*-value
Depressive symptoms^a^						
Normal	−			−		
Mild	−3.224	3.386	0.343	−0.492	2.654	0.853
Moderate to severe	−9.993	4.252	0.021	−4.671	2.916	0.112
Age	−1.780	2.284	0.437	−5.757	1.937	0.004
Body mass index	0.028	0.412	0.946	0.253	0.316	0.426
At-risk drinking^b^	−4.101	2.684	0.129	−6.494	2.975	0.031
Regular exercise(≥1/week)	6.185	2.804	0.029	3.932	2.604	0.134
Age at menarche				−2.532	0.753	0.001
Duration of breastfeeding						
None				−		
1−11 months				−0.704	2.632	0.790
≥12 months				−4.535	2.907	0.121
Adjusted *R*-square	0.071			0.160		

PE, parameter estimate; SE, standard error.

K-BDI, the Korean version of the Beck Depression Inventory.

a Depressive symptoms were classified as normal (K-BDI <10), mild (K-BDI 10−15), and moderate to severe (K-BDI ≥16).

b At-risk drinking was defined as consuming an average of more than 2 drinks/day for men and 1 drink/day for women.

## Discussion

In the present study examining the association between depressive symptoms and bone density in apparently healthy Korean men and women aged 29 to 32 years, K-BDI scores were negatively correlated to the SI and the participants with more severe depressive symptoms tended to have a lower SI in men. In women, similar trends were shown, but were statistically insignificant. Although there is little evidence in young adults, our results are consistent with a previous study in different ethnic groups: in a study with 5,171 non-Hispanic white, African-American, and Mexican-American men and women aged 20 to 39 years participated in the Third National Health and Nutrition Examination Survey, major depressive episode or dysthymia assessed by the Diagnostic Interview Schedule was associated with lower BMD measured by DXA in men, but not in women [11]. In this study, gender difference was explained by higher prevalence rates for depression and dysthymia in women compared to men. The authors also suggested that impact of depression on physical activity or diet might be more severe in men than women [11]. For premenopausal women, however, several studies with relatively small sample size are available. Although the results were inconsistent, the majority demonstrated an inverse association between depression and BMD [7]–[9], [20]–[23]. In a cross-sectional study with 25 premenopausal women with MDD (mean age: 30.8±8.4 years) and 15 normal women (mean age: 31.2±7.9 years) in Turkey, depressed women had a significantly lower BMD at the lumbar spine and femur [20]. In a case-control study with 73 premenopausal women aged 30 to 49 years with unipolar depression and age- and osteoporosis risk factor-matched 47 healthy women in Serbia, women with depression had a significantly lower BMD at the lumbar spine and femoral neck [21]. In another case-control study, BMD at the lumbar spine and femur of 42 premenopausal women (mean age: 35.4±7.5 years) diagnosed with depression were compared to those of age- and BMI-matched 42 healthy women (mean age: 36.7±6.7 years) in Turkey [22]. In this study, no significant association was shown [22]. In a 36-month prospective study with 92 premenopausal women between the ages of 21 and 45 years old from the U.S., women with MDD tended to have a lower BMD measured by DXA than the 44 healthy controls [23]. Among Asian populations, a few studies have been conducted in older adults. In a study with 1,999 Hong Kong Chinese men aged 65 to 92 years, depressed patients assessed using the Geriatric Depression Scale (n = 169) had a lower BMD measured by DXA than controls (n = 1,830) [24]. Another study in 2,611 community-dwelling Chinese aged 55 and older in Singapore demonstrated that depressed patients assessed using the Geriatric Depression Scale (n = 347) had a higher risk of having osteoporosis, but the statistical significance was attenuated after adjusting for covariates [25]. In addition, a study with 932 community-dwelling Korean men and women aged 60 to 80 years had results consistent with the present study; depressive symptoms measured by the K-BDI showed a significantly negative correlation to the SI in older men, but not in women [26].

Both osteoporosis and depression are a growing health care burden [2], [27], therefore, preventing osteoporosis and depression is critically important. For young adults continuing to adolescent, achieving a higher peak bone mass is an important factor to prevent osteoporosis later in life. Though both genetic and environmental factors are known to contribute to peak bone mass, genetic factors such as heredity and sex are not modifiable. Therefore, preventive approaches should be focused on modifiable, environmental factors. Currently, adequate nutritional intakes (especially calcium intake), exercise, hormonal factors, and exposure to behavioral risk factors are known determinants of peak bone mass [3], [28]. In addition to them, if depressive symptoms negatively affect bone mass in young adults, prevention or treatment of depression in young adults might be crucial.

Despite the growing evidence of the link between depression and osteoporosis, the biologic mechanism remains unclear. Evidence to support that depression may be a risk factor for osteoporosis includes alteration in the adrenergic axis and the hypothalamic-pituitary-adrenal axis [7], [29]. Experimentally depressed mice displayed impaired bone formation and an increase in bone norephinephrine levels [30]. In these mice, the fact that β-adrenergic antagonist blocked bone loss suggested that the sympathetic nervous system mediate the depression-triggered bone loss [30]. Glucocorticoids, which are known to correlate to depression [31], also suppress bone formation and increase bone resorption via inhibiting osteoblastogenesis and promoting apoptosis of osteoblasts and osteocytes [32]. Another possible bone-brain connection is related to serotonin [33] of which the target tissues include the brain and bone [34]. Although the effect of serotonin on bone differs depending on its origin, serotonin activity in the brain favorably influences bone mass and precedes duodenum-derived serotonins that inhibit bone formation [35]. As serotonins can be stored in or released by platelet during the clotting process, a reduced uptake of serotonin in platelets and neurons during depression possibly influences free serotonin on bone mass [33]. In practice, depressed patients treated with selective serotonin reuptake inhibitor displayed decreased BMD [36]. Another possible mechanism is that depression and low BMD may share common risk factors or common pathways. Poor health behavior such as smoking, increased alcohol consumption, low physical activity, or comorbidity was associated with both depression and BMD [8]. Also low diet quality [37], [38] or changes in sleeping patterns [39] in depressed patients may also affect BMD via inadequate nutrition [40], [41], impaired insulin sensitivity [42], or alteration of neurotransmitter receptor systems such as serotonin, norephinephrine or glucocorticoids [43], [44]. Pro-inflammatory markers (e.g., interleukin-6 or C-reactive protein) were elevated in depressed patients [45], [46] and were also associated with low BMD [46], [47]. In addition, sex hormones including estrogen and testosterone were related to both depression [48], [49] and bone formation and turnover [50]. We therefore controlled for current smoking, at-risk drinking, and regular exercise as covariates in the multivariate-adjusted analyses. With regards to comorbidity, our participants were young and relatively healthy with no reported cancer, stroke, or ischemic heart disease. Further, we confirmed that BMI, blood pressure, serum levels of C-reactive protein, cholesterol, and glucose were not significantly different among participants across the severity of depressive symptoms in this study population (Table 1).

In this study, sex difference existed in the results: the negative association between depressive symptoms and the SI was observed in men, but not in women. According to a study from a nationwide survey in Korea, BMD significantly decreased from the third decade of life in men. Meanwhile, in women, BMD plateaued until the fifth decade and rapidly declined peri- and post-menopause [51]. Therefore, early bone loss occurs more frequently in men because male bone may be more vulnerable to factors such as depressive symptoms than female bone. Additionally, sex hormones, especially estrogen in women, are one of the most important determinants of bone health [3]. In fact, age at menarche was negatively associated with the SI in women (Table 2). It can be explained that women with earlier menarche might be exposed to more estrogen, therefore, strong protective effects of female sex hormones might overcome the negative effects of psychological stress on bone density.

This study has several limitations. First, the sample size of the present study was relatively small. However, considering that age is one of the most determining factors for bone density, the fact that study was performed in a focused age group, between ages of 29 and 32 years, could be an added strength. In addition, this study investigated the association between depressive symptoms and bone density in both Korean men and women; therefore, our study might provide evidence for young Asian populations. Second, the K-BDI is a well-known screening tool to detect depression in normal populations, so K-BDI scores might provide the information of having depressive symptoms in this study population. However, the K-BDI is not a diagnostic tool for depression; therefore, the study results cannot be generalized to patients with clinically diagnosed depression. Third, we measured the SI using a quantitative ultrasound device. Although the SI at the os calcis correlates with BMD by DXA which is the current gold standard method [14], [52], further confirmation with DXA is needed. Fourth, diet and sleeping patterns, which may be different in depressed participants, were not evaluated in this study. Since insufficient nutrition and a low sleep quality may affect BMD, residual confounding possibly exists. Fifth, the mean age of participants was 31 years. Thereby, their bone masses may be close to their peaks. However, we did not serially measure their SI, so the effect of depressive symptoms or other covariates on peak bone mass could not be evaluated in this study. Finally, due to the cross-sectional design and limited information on biological mechanisms, we cannot conclude a causal relationship between depressive symptoms and the SI.

In conclusion, the present study demonstrated a negative association between depressive symptoms and the SI in apparently health young Korean men, but not in women. Further studies are necessary to evaluate the effect of depressive symptoms on lower bone density in young adulthood and the impact of depression on developing osteoporosis or osteoporotic fractures later in life.
